# Supplementary material for: Status of psychological health of students following the extended university closure in Bangladesh: Results from a web-based cross-sectional study
Source: PLOS Glob Public Health. 2022 Mar 31;2(3):e0000315. doi: 10.1371/journal.pgph.0000315 (PMC10021959; doi:10.1371/journal.pgph.0000315)
Supplement: S1 Table — (DOCX) [file pgph.0000315.s001.docx]

**Appendix**

**Table S1: The PHQ-9 (PHQ = Patient Health Questioner) and GAD-7 (GAD = Generalized Anxiety Disorder) items to investigate the major depressive disorder and generalized anxiety disorder among the university students following the extended COVID-19 lockdown in Bangladesh.**

| **PHQ_1: Little interest or pleasure in doing things?** | Not at all | 83 | 17.8 |
| --- | --- | --- | --- |
|  | Several days | 195 | 41.9 |
|  | More than half the days | 67 | 14.4 |
|  | Nearly everyday | 120 | 25.8 |
| **PHQ_2: Feeling down, depressed, or hopeless?** | Not at all | 93 | 20.0 |
|  | Several days | 206 | 44.3 |
|  | More than half the days | 66 | 14.2 |
|  | Nearly everyday | 100 | 21.5 |
| **PHQ_3: Trouble falling or staying asleep, or sleeping too much** | Not at all | 107 | 23.0 |
|  | Several days | 153 | 32.9 |
|  | More than half the days | 58 | 12.5 |
|  | Nearly everyday | 147 | 31.6 |
| **PHQ_4: Feeling tired or having little energy?** | Not at all | 125 | 26.9 |
|  | Several days | 198 | 42.6 |
|  | More than half the days | 55 | 11.8 |
|  | Nearly everyday | 87 | 18.7 |
| **PHQ_5: Poor appetite or overeating?** | Not at all | 187 | 40.2 |
|  | Several days | 146 | 31.4 |
|  | More than half the days | 44 | 9.5 |
|  | Nearly everyday | 88 | 18.9 |
| **PHQ_6: Feeling bad about yourself - or that you are a failure or have let yourself or your family down?** | Not at all | 145 | 31.2 |
|  | Several days | 164 | 35.3 |
|  | More than half the days | 44 | 9.5 |
|  | Nearly everyday | 112 | 24.1 |
| **PHQ_7: Trouble concentrating on things, such as reading the newspaper or watching television?** | Not at all | 195 | 41.9 |
|  | Several days | 123 | 26.5 |
|  | More than half the days | 48 | 10.3 |
|  | Nearly everyday | 99 | 21.3 |
| **PHQ_8: Moving or speaking so slowly that other people could have noticed? Or The opposite - being so fidgety or restless that you have been moving around a lot more than usual?** | Not at all | 290 | 62.4 |
|  | Several days | 99 | 21.3 |
|  | More than half the days | 45 | 9.7 |
|  | Nearly everyday | 31 | 6.7 |
| **PHQ_9: Thoughts that you would be better off dead, or of hurting yourself in some way?** | Not at all | 308 | 66.2 |
|  | Several days | 82 | 17.6 |
|  | More than half the days | 27 | 5.8 |
|  | Nearly everyday | 48 | 10.3 |
| **GAD_1: Feeling nervous, anxious, or on edge?** | Not at all | 144 | 31.0 |
|  | Several days | 208 | 44.7 |
|  | More than half the days | 40 | 8.6 |
|  | Nearly everyday | 73 | 15.7 |
| **GAD_2: Not being able to stop or control worrying?** | Not at all | 214 | 46.0 |
|  | Several days | 145 | 31.2 |
|  | More than half the days | 44 | 9.5 |
|  | Nearly everyday | 62 | 13.3 |
| **GAD_3: Worrying too much about different things?** | Not at all | 76 | 16.3 |
|  | Several days | 186 | 40.0 |
|  | More than half the days | 58 | 12.5 |
|  | Nearly everyday | 145 | 31.2 |
| **GAD_4: Trouble relaxing?** | Not at all | 168 | 36.1 |
|  | Several days | 175 | 37.6 |
|  | More than half the days | 51 | 11.0 |
|  | Nearly everyday | 71 | 15.3 |
| **GAD_5: Being so restless that it is hard to sit still?** | Not at all | 217 | 46.7 |
|  | Several days | 138 | 29.7 |
|  | More than half the days | 53 | 11.4 |
|  | Nearly everyday | 57 | 12.3 |
| **GAD_6: Becoming easily annoyed or irritable?** | Not at all | 96 | 20.6 |
|  | Several days | 166 | 35.7 |
|  | More than half the days | 73 | 15.7 |
|  | Nearly everyday | 130 | 28.0 |
| **GAD_7: Feeling afraid as if something awful might happen?** | Not at all | 159 | 34.2 |
|  | Several days | 147 | 31.6 |
|  | More than half the days | 62 | 13.3 |
|  | Nearly everyday | 97 | 20.9 |
