# Supplementary material for: Status of psychological health of students following the extended university closure in Bangladesh: Results from a web-based cross-sectional study
Source: PLOS Glob Public Health. 2022 Mar 31;2(3):e0000315. doi: 10.1371/journal.pgph.0000315 (PMC10021959; doi:10.1371/journal.pgph.0000315)
Supplement: Attachment — Submitted filename: Responses to reviewer.docx [file pgph.0000315.s003.docx]

**Point by point reviewers’ responses**

**Manuscript Title:** Effects of extended university closure on students’ psychological health in Bangladesh: Results from a web-based cross-sectional study

**Manuscript ID:** PGPH-D-21-00846

Additional Editor Comments (if provided):

Thank you for sharing this article, I agree with the reviewer comments about revising the methods and more heavily referencing the article. I also recommend conducting a close reading and revision of the article for grammar, language, and clarity. Please also revise table 1 and only highlight pertinent variables such as the GAD scores etc. and not the questions that fed into GAD scores (these could potentially be shared in an appendix instead) similarly for PHQ.

Authors’ responses: Thank you very much for considering our manuscript for its further revision. We have carefully studied your comments and reviewers’ comments/suggestions and improved our manuscript accordingly. We have also carefully revised and improved the grammar of the manuscript. Also, we have eliminated all the unintentional typos errors. According to your suggestions, we have also corrected table 1 and moved the PHQ-9 and GAD-7 items into the supplementary file (Table S1).

**Reviewers' comments:**

**Comments to the Author**
1. Does this manuscript meet PLOS Global Public Health’s [publication criteria](https://journals.plos.org/globalpublichealth/s/criteria-for-publication)? Is the manuscript technically sound, and do the data support the conclusions? The manuscript must describe methodologically and ethically rigorous research with conclusions that are appropriately drawn based on the data presented.

Reviewer #1: Yes

Reviewer #2: Partly

Authors’ responses: Thank you very much to the reviewers for reviewing the manuscript and providing your kind feedback. We appreciate your feedback.

2. Has the statistical analysis been performed appropriately and rigorously?

Reviewer #1: Yes

Reviewer #2: No

Authors’ responses: Thank you very much for your kind feedback.

3. Have the authors made all data underlying the findings in their manuscript fully available (please refer to the Data Availability Statement at the start of the manuscript PDF file)?

The [PLOS Data policy](https://journals.plos.org/globalpublichealth/s/data-availability) requires authors to make all data underlying the findings described in their manuscript fully available without restriction, with rare exception. The data should be provided as part of the manuscript or its supporting information, or deposited to a public repository. For example, in addition to summary statistics, the data points behind means, medians and variance measures should be available. If there are restrictions on publicly sharing data—e.g. participant privacy or use of data from a third party—those must be specified.

Reviewer #1: Yes

Reviewer #2: Yes

Authors’ responses: Thank you very much for your kind feedback.

4. Is the manuscript presented in an intelligible fashion and written in standard English?

PLOS Global Public Health does not copyedit accepted manuscripts, so the language in submitted articles must be clear, correct, and unambiguous. Any typographical or grammatical errors should be corrected at revision, so please note any specific errors here.

Reviewer #1: No

Reviewer #2: No

Authors’ responses: Thank you for your feedback regarding the English status of the manuscript. We have revised and eliminated any unintentional grammar and typos errors of the manuscript.

5. Review Comments to the Author

**Reviewer #1:** Overall comment: This is an important piece of work and while the study appears to be sound, the language is unclear, making it difficult to follow. I advise the authors work with a writing coach or copyeditor to improve the flow and readability of the text.
Authors’ responses:

Thank you very much. We appreciate your point. We, all the authors of the manuscript, worked hard to revise the manuscript and improved its language with a good flow of writings. Besides, we have eliminated all the grammar and typos errors with the help of an English expert.

Abstract: Is well written, however the methods indicate univariate, bivariate, and multivariate analyses were conducted, but no specific mention in the results of the abstract.

Authors’ responses:

Thank you very much for the point. We have stated the following lines in the abstract part for improving the method and specified results according to your suggestions

*Frequency and percentage distribution as univariate, chi-square (χ^2^) test as bivariate, and logistic regression as multivariate analyses were applied to investigate the prevalence of depression and anxiety and their associated various sociodemographic factors. (Lines: 38-40)*

*The students from the private universities were two times and 2.7 times more depressed and anxious, respectively than the students from the public universities. In addition, the students who became incomeless had significantly more anxiety (adjusted odds ratio [AOR] = 1.711; p = 0.018) than those who did not lose income source during the COVID-19 lockdown. (Lines: 43-47)*

Introduction: Should be more focused on the Bangladeshi situation. Recommendation to reduce, especially text around global trends.

Authors’ responses:

Thank you very much for your comments and suggestions. We have shortened the international data and kept focused on the Bangladeshi situation. For example, we have deleted lines 89-91 and lines 105 (previous version). We have added some BD data for example, lines 109-115 as follows: (revised version)

*Consequently, several mental health complications have arisen extremely among Bangladeshi university students. It has recently been reported that around 50%-60% of Bangladeshi university students were suffering from underprivileged online education, severe session jam phobia, and extreme psychological distress [22]. In a systematic review, Mamun et al. [23] stated that 46.92% to 82.4%, 26.6% to 96.82%, and 28.5% to 70.1% of Bangladeshi students faced mild to severe symptoms of depression, anxiety, and stress, respectively during COVID-19 lockdown.* (Lines: 109-115)

Methods: Did you collect data on previous mental health condition before COVID-19? Or if students had COVID-19 or exposure to COVID-19? The literature suggests these are important covariates/confounders.

Authors’ responses:

Thank you very much for your nice questions. All the students who were never clinically diagnosed mentally distorted before COVID-19 were included in the study. The students who were suffering mental health complications since before COVID-19 were excluded from the study. (Lines: 130-131)

Results: Are well described.

Authors’ responses: Thank you very much for your kind comments.

Discussion: Is quite lengthy and would benefit from a read through to reduce. Limitations were well addressed. Should include a point on previous mental health conditions and exposure/COVID-19 + if these data weren't collected. This could alter findings. Should also put this study in context. Are there any recommendations for future research, programs or policy based on your findings?

Authors’ responses: Thank you very much for your comments and suggestions. We have carefully revised the discussion part and reduced more than 120 words. Additionally, we have corrected in some places and improved the discussion part. We did not include the students in the survey who were associated with mental health problems during pre-COVID-19 time. We also added a new point regarding the implications and future research according to your suggestions as follows:

***Implications and future research***

*The current study has investigated two major psychological disturbances of Bangladeshi university students following the prolonged COVID-19 lockdown. The results obtained in the study might be representative of the university students’ current mental health complications of the other countries having equivalent socioeconomic and cultural characteristics. The research findings will help the policymakers, government, educationalists, and university authorities determine the prevalence of these psychological disorders (depression and anxiety) and their triggering factors. The current statistics will also help the administrators to resolve the severe mental health complications of the university students by taking several effective measures, like implementing professional counseling, economic and job support, and ensuring smooth educational activities. However, several studies need to be conducted to compare the prevalence of the mental health complications of university students with other age groups of people or professionals. Besides, the new normal trends like online classes and online exam systems are equally efficacious for all learners with distinct socioeconomic conditions.* (Lines; 349-362)

Referencing: Some inconsistencies and sentences that should have a citation.

Authors’ responses: Thank you very much. We have revised and corrected the text and reference sections. We have also added some citations where the previous version missed citations in some places.

**Reviewer #2:** Please make major corrections. I have made comments inside the manuscript. please check. Study design, rationale, objectives, tools, statistical analysis are not well- described. English is poor. Tools are used in clinical set-up.

Questions and comments from PDF:

**Abstract:**

Reviewer’s comment: cross-sectional pilot study? what type of study is this???

Authors’ responses: Thank you very much for the question. It should have been cross-sectional study. We have corrected it to cross-sectional study.

Reviewer’s comment: what type of study design this is???

Authors’ responses: This study also can be referred as web-based study as we conducted study using google form. We have also found some similar studies that were referred as web-based studies. However, we corrected the line to avoid confusion to the readers.

Reviewer’s comment: not reflected in the result part

Authors’ responses: Thank you for the comments. Univariate, bivariate, and multivariate analysis are reflecting in the result section. We conducted frequency and percentage distribution as univariate analysis, chi-square test as bivariate analysis, and logistic regression as multivariate analysis which was represented in the figure.

Reviewer’s comment: what type of measures?

Authors’ responses: In conclusion of abstract we have mentioned some measures that can be taken into consideration to relief the students from psychological distress. To keep the abstract concise, we didn’t mention them in abstract.

**Introduction:**

Reviewers’ comment: 1. English is poor. 2. Not adequate literatures 3. absent of proper justification

Authors’ responses: Thank you for the comments. As per your concern, we have thoroughly read the whole text and improved its language. We have also eliminated any unintentional grammar and typos errors. Additionally, we have carefully studied the current literature in Google Scholar and PubMed & improved the introduction and justification of the study as follows:

*Consequently, several mental health complications have arisen extremely among Bangladeshi university students. It has recently been reported that around 50%-60% of Bangladeshi university students were suffering from underprivileged online education, severe session jam phobia, and extreme psychological distress [17]. In a systematic review, Mamun et al. [22] stated that 46.92% to 82.4%, 26.6% to 96.82%, and 28.5% to 70.1% of Bangladeshi students faced mild to severe symptoms of depression, anxiety, and stress, respectively during COVID-19 lockdown.*

*Several socioeconomic factors associated with negative emotions exacerbated by the extended COVID-19 lockdown have intensely impacted the mental health conditions of all ages, particularly students, due to the university closure for a prolonged time [17,23]. Ela et al. [21] reported that the graduation delay had been mainly occurred due to the lengthy university closure that has significantly increased various mental health complications, including depression and anxiety, among the tertiary level of students in Bangladesh. Besides, Bangladesh was suffering from the 2^nd^ stream of COVID-19 outbreak from the last months of 2020 that has worsened the students' mental complications [24].* (Lines: 109-123)

**Methods:**

Reviewer’s comment: describe your design clearly

Authors’ responses: Thank you very much for the suggestions. We have clearly described the study and several new lines have been added as follows:

*The questionnaire was segmented into three sections where section A consisted of the sociodemographic information of the participants, and sections B and C were designed with depression and anxiety-related questions. After the initial drafting of the questionnaire in English, it was then translated into in Bangla version. Then the questionnaire was checked and validated by a bilingual medical expert through a forward and backward translation process [25].* (Lines: 133-138)

*The students who were never clinically diagnosed with mental distortion before COVID-19 were included in the study.* (Lines: 141-142)

Reviewer’s comment: Both are used in clinical settings. How did you use these tools here?

Authors’ responses: Thank you very much for the comment and question. Although these scales were first suggested in the clinical set-up; several studies used these scales for online surveys. They studied the consistency of the scales online set-up and found them reliable to use for online surveys. In our study, we also measured Cronbach’s alphas to measure the reliability of the scales for our study design. We found high values of Cronbach’s alphas that implies the scales are reliable for our study.
