# Supplementary material for: Status of psychological health of students following the extended university closure in Bangladesh: Results from a web-based cross-sectional study
Source: PLOS Glob Public Health. 2022 Mar 31;2(3):e0000315. doi: 10.1371/journal.pgph.0000315 (PMC10021959; doi:10.1371/journal.pgph.0000315)
Supplement: Attachment — Submitted filename: Responses to reviewer comments.docx [file pgph.0000315.s004.docx]

**Point by point reviewers’ responses**

**Manuscript Title:** Effects of extended university closure on students’ psychological health in Bangladesh: Results from a web-based cross-sectional study

**Manuscript ID:** PGPH-D-21-00846.R1

**Review comments PGPH-D-21-00846R1**

Initially I supossed that review this paper would be easy for me because I just have finished a study analysing self-harm behaviours during the COVID-19 pandemic. All the bibliography I reviewed indicated an increased anxiety and depressive symptoms severity in the population (including universitary student). To know the impact of COVID-19 control measures is necessary to avoid the mistake in possible future pandemic contexts. However, I hope the authors apologize me for the comment, but I thinks that the manuscript need to be revised.

Authors’ responses: Thank you very much for spending your valuable time reviewing the manuscript and providing your expert opinions and suggestions. Your comments and suggestions helped improve the manuscript. We have carefully studied all the comments and made several revisions throughout the manuscript, which we hope will merit your approval.

In my opinion, the main problem with the manuscript is that it does not reflect a clear understanding of what the scientific method entails. I try to explain my considerations:

First of all, I am disconcerted with the title. When I read “Effects of extended university closure on students' psychological health in Bangladesh: Results from a web-based cross-sectional study”, I supposed that authors would test how the university closure and no other factors affected to a sample of university students. However, the cross-sectional design of the study and the text of the manuscript indicated that authors, only has study the relationships of sociodemographic variables with anxiety and depressive symptoms in a context that included the university closure, but in any case the effect of closure. Or can be the authors sure with this study that was the university closure the cause of symptomatology and not other factors implicated in this chaoatic context of COVID-19?

Authors’ responses: Thank you very for your comments and concerns. In order to remove the confusion, we have changed the title as follows:

*“Status of psychological health of students following the extended university closure in Bangladesh: Results from a web-based cross-sectional study” (Lines: 1-2)*

In line of the above mentioned, I sincerely recommended to authors read Kar Popper. None study can demostrate anythings, only it could support some hypothesis. Rewrite the paper and please review sentences along the manuscript, as the first one of discussion “the present study demostrated…. (line 256)”. Related to this, I considered it is important to rewrite the hypothesis:

Authors’ responses: Thank you very for your suggestions. We have revised and rewritten the lines as follows:

*“The present study reported the prevalence of anxiety and depression by 53.5% and 54.6%, respectively, among university students, closely resembling the previous study in Bangladesh.” (Lines: 257-258)*

Therefore, in this paper, we aim to examine the influences of various socio-demographic factors potentially responsible for enhancing several psychological problems in terms of depression and anxiety endured by various university students in Bangladesh *due to the extended shutdown of their educational institutions*.

Can authors be sure about the only causation of educational shutdown in Bangladesh of anxiety and depression in universitary students? Or are there a complex multivariables relationships relationships with anxiety and depression during COVID-19 pandemic? All our studies results are important to us, but we have to be aware of what explains certain results of the complexity of human behavior.

Authors’ responses: Thank you immensely for your questions. The students were indeed suffering from various mental health complications due to the educational instability during the COVID-19 university closure. However, only the educational institution's shutdown may not be the only factor for these psychological complications. So, we have revised the lines as follows:

*Therefore, in this paper, we aim to investigate the influences of various socio-demographic factors potentially responsible for enhancing several psychological problems in terms of depression and anxiety endured by university students in Bangladesh due to the extended COVID-19 shutdown. (Lines: 124-127)*

On other side, about the introduction, in my opinion, the authors could still improve the introduction. The first parragraph (lines 58-71) adecuately introduce the context (may be also to long, but adecuately), the second one (lines 72-80) also adecuately describe the relationships of COVID-19 control measures with mental health. However, in the last one I missed important evidence that shows results about the relationship between COVID-19 control measures and mental health. I recommend to the authors review some of the following studies:

Racine et al., 2021 doi:10.1001/jamapediatrics.2021.2482

Pieh et al., 2021 doi:10.1001/jamanetworkopen.2021.14866

Panchal et al., 2021 doi.org/10.1007/s00787-021-01856-w

…. And maybe the following ones could help the authors to improve the importance of theirs aims:

Tuquet et al., 2021 doi.org/10.1016/S0140-6736(21)02221-2

Cousien et al., 2021 doi:10.1001/jamanetworkopen.2021.28611

Robillard et al., 2021 doi.org/10.1016/j.psychres.2021.114152

Authors’ responses: Thank you very much for your nice obervations and suggestions. We have revised the first paragraph and extended the evidence focusing the relationship of mental health and covid1-9 control mesaures as follows:

*Several studies reported that the lockdown and associated restrictions (e.g., online schooling, social distancing) imposed due to the COVID-19 pandemic has resulted in mental health impairments [9-11]. Young people are more likely to be more sufferers of psychological disorders than adults, and they are more vulnerable to the adverse effects of social isolation, including educational institution closure [11]. A meta-analysis on 29 studies (80 979 youth) showed that the global prevalence of clinically increased child and adolescent depression and anxiety was 25.2% and 20.5%, respectively [9]. Besides, Humer et al. [10] reported that school reopening and reduced social distancing measures were associated with improved mental health conditions among high school students.* (Lines: 171-179)

Ref:

9. Racine N, McArthur BA, Cooke JE, Eirich R, Zhu J, Madigan S. Global Prevalence of Depressive and Anxiety Symptoms in Children and Adolescents During COVID-19: A Meta-analysis. JAMA Pediatr. 2021 Nov 1;175(11):1142-1150. doi: 10.1001/jamapediatrics.2021.2482. PMID: 34369987; PMCID: PMC8353576.
10. Humer E, Dale R, Plener PL, Probst T, Pieh C. Assessment of Mental Health of High School Students 1 Semester After COVID-19-Associated Remote Schooling Measures Were Lifted in Austria in 2021. JAMA Netw Open. 2021 Nov 1;4(11):e2135571. doi: 10.1001/jamanetworkopen.2021.35571. PMID: 34807262; PMCID: PMC8609406.
11. Panchal U, Salazar de Pablo G, Franco M, Moreno C, Parellada M, Arango C, Fusar-Poli P. The impact of COVID-19 lockdown on child and adolescent mental health: systematic review. Eur Child Adolesc Psychiatry. 2021 Aug 18:1–27. doi: 10.1007/s00787-021-01856-w. Epub ahead of print. PMID: 34406494; PMCID: PMC8371430.

The third introduction paragraph (lines 81-89), also in my opinion, is the best of the introduction. It clearly introduduce the context of the study aim. However, the fouth paragraph is hard to read. Change it for a clear abstract of the current paragraph, without incessesary detailed information (ex. economical problem, could be used to include to much of the information the authors detalled in the parragraph) wil improve it considerabily. Despite the previous comment, the last sentences of the paragraph (lines 109-115) is a good final for the above mentioned abstract.

Authors’ responses: Thank you very much for your comments and appreciation. As per your instruction, we have deleted the lines 90-101 (PGPH-D-21-00846.R1).

Finally, the last paragraph also needs to be improved. The beginning is redundant, but it includes new information that might be interesting to include in the previous one, where mental health problems in university students have already been discussed. This paragraph will be improved if the authors focused it in the aim (what does various socio-demographic factors means?). What is the author hypothesis?

Authors’ responses: Thank you very much for the comments. According to your comments, we have shifted the intial information of the last paragraph into the previous paragraph. The study covariates/socio-demographic factors were evaluated to find out the association with the students’ anxiety and depression. The study covariates or socidemigraphics were specificed in the methods sections.

Please review what does “by various university students” mean? (line 125) Review the study aim.

Authors’ responses: Thank you very much for the instuction. “by various university studnets” mean the students from “public”, “private” and others category students. We have revised the study aim as follows:

*Therefore, in this paper, we aim to investigate the influences of various socio-demographic factors potentially responsible for enhancing several psychological problems in terms of depression and anxiety endured by university students in Bangladesh due to the extended COVID-19 shutdown.* (Lines: 119-122)

Altough the introduction comments could easier improve the manuscript, the methods section did not let me to undestrand what and how authors did the study. I had to read several times to understand it, search the references to know what had been done and although the authors say that they used logistic regression, I was left without knowing what method they used to analyze the effect of covariates. Could it be interesting to include the variables by steps? However, the worse problem is that, altough I looked for information about the questionnaire (I need to know how the variables were measures to understand the results) and I diagonally review the references cited by the authors (Hossain et al., 2021 doi.org/10.1016/j.heliyon.2021.e07388; Uddin et al., 2018 doi:10.1136/bmjopen-2018-022967), I could not solve my doubt. Therefore, if I need to make so much effort to know what the authors explain to me, I consider that the method is not well explained. For example, I would like to know how the authors measured when the symptoms appeared? Was the duration of symptoms measured in any way? On the other hand, why didn't they use the total symptom score instead of the cut-off point?...

Moreover, Authors explain the questionnaire in study desing and sampling section, where indicated that depression and anxiety-related questions were measured in section B and C, reference 25, and after in the covariates section in assessment of depression and anxiety, authors explaing how anxiety and depression were measured. It could be reorganized to facilitate the reading? Finally, one of the reference about the questionnaries used in the present study PHQ-9 and GAD-7, aimed to validate the K-10. Confusing!!!!! I could not lost more time reviewing the method. Authors should make the article easier to read it is important to know how were measures the variable to understand the results!

Authors’ responses: Thank you very much for your comments and criticism regaridng the method sections. The questionnniare was designed according to the Islam et al. 2020 (doi: [10.1371/journal.pone.0238162](https://doi.org/10.1371/journal.pone.0238162)). As per your concern, we have revised and changed the reference. We have also expalined the variables through two points (“Covariates” and “Assessment of depression and anxiety”). Hope your confusion has been cleared regaridng the methods. Besides, considering different characteristics, we could consider the total score that will give information on how distress or anxiety increases. But, our study goal was to see how diverse respondents' backgrounds are associated with severely depressed and anxious individuals.

Finally, after I read the covariates section, and due to the lack of clarity about the measures in the methods section, I arribe to the conclusion that author can not tested theirs aim. **The authors only can test the relationships of sociodemgraphic variables with anxiety and depression during COVID pandemic in universitary students**, but not afirm that anxiety and depression was due to the extended shutdown of their educational institutions. For that purpose, authors would need at least one case-control study, not a correlational one.

Authors’ responses: Thank you very much for your comments. The previous title of the study was unclear to you. As we did not investigate the direct effects of university closure on the depression and anxiety in the study, we have changed the title and hopefully, the current title of the manuscript will clear the confusion.

Related with the results section, It will be interesting to considered the χ2 test results interpretation under the considerations of the influence of n subsamples in some significant results. Moreover, in the results of logistic regression author wrote “During multivariate analysis, binary logistic regression was applied for exploring the significantly influencing variables after adjusting other parameters that may accelerate the psychological distress conditions of university students in Bangladesh” Please, I need information about the regression analysis procedure and the results for the “influencing variables” and for “adjusted variables”. I do not understand the prior sentence without these information.

Authors’ responses: Thank you for your comments. When we included several independent variables in logistic regression, the Odds Ratio (OR) was adjusted OR. It finds the effect of a particular variable keeping all other independent variables at a fixed level, which we have tried to say in this sentence.

Based on the previously commented I thinks also that discussion must be improved. Authors must review the extensively bibliography about factors influencing anxiety and depressive symptoms, specially during the COVID-19 pandemic and cleary considered the limitation of a correlational study when discussing theirs results.

Authors’ responses: Thank you for your comments. We have revised the every factor significantly assoctaed with depression and anxiety was described with suitable previous references. Several more expalnations were added accoridng to your suggestions; for example,

*……Before the current COVID-19 pandemic, the prevalence of anxiety and depressive symptoms among youth was 11.6% and 12.9%, respectively [9]. (Lines 257-258)*

*……The students away from families were more mentally distressed than the students who were passing their time with families, and the hypothesis was endorsed by previous evidence [47]. (Lines: 315-317)*

*……Many studies reported that the economic crisis significantly accelerated the increase of common mental health complications [48, 49]. The prevalence of major depressive disorder was found to be increased 8.2% in 2011 from 3.3% in 2008 after the 2009 economic crisis in Greece [50]. Study participants expressing serious financial hardship were most at risk of developing the symptoms of major depressive disorder [48, 50]. (Lines: 326-331)*

9. Racine N, McArthur BA, Cooke JE, Eirich R, Zhu J, Madigan S. Global Prevalence of Depressive and Anxiety Symptoms in Children and Adolescents During COVID-19: A Meta-analysis. JAMA Pediatr. 2021 Nov 1;175(11):1142-1150. doi: 10.1001/jamapediatrics.2021.2482.
47. Horigian VE, Schmidt RD, Feaster DJ. Loneliness, Mental Health, and Substance Use among US Young Adults during COVID-19. J Psychoactive Drugs. 2021 Jan-Mar;53(1):1-9. doi: 10.1080/02791072.2020.1836435.
48. Frasquilho D, Matos MG, Salonna F, Guerreiro D, Storti CC, Gaspar T, Caldas-de-Almeida JM. Mental health outcomes in times of economic recession: a systematic literature review. BMC Public Health. 2016 Feb 3;16:115. doi: 10.1186/s12889-016-2720-y.
49. COVID-19 Mental Disorders Collaborators. Global prevalence and burden of depressive and anxiety disorders in 204 countries and territories in 2020 due to the COVID-19 pandemic. Lancet. 2021 Nov 6;398(10312):1700-1712. doi: 10.1016/S0140-6736(21)02143-7.
50. Economou M, Madianos M, Peppou LE, Patelakis A, Stefanis CN. Major depression in the era of economic crisis: a replication of a cross-sectional study across Greece. J Affect Disord. 2013 Mar 5;145(3):308-14. doi: 10.1016/j.jad.2012.08.008.
